# Co-enrolment of Participants into Multiple Cancer Trials: Benefits and Challenges

**DOI:** 10.1016/j.clon.2017.02.014

**Published:** 2017-07

**Authors:** F.H. Cafferty, C. Coyle, S. Rowley, L. Berkman, M. MacKensie, R.E. Langley

**Affiliations:** ∗ MRC Clinical Trials Unit at UCL, London, UK; † NCRI Consumer Liaison Group, London, UK; ‡ Independent Cancer Patient Voices, London, UK

**Keywords:** Adjuvant, aspirin, cancer, co-enrolment, randomised controlled trial

## Abstract

Opportunities to enter patients into more than one clinical trial are not routinely considered in cancer research and experiences with co-enrolment are rarely reported. Potential benefits of allowing appropriate co-enrolment have been identified in other settings but there is a lack of evidence base or guidance to inform these decisions in oncology. Here, we discuss the benefits and challenges associated with co-enrolment based on experiences in the Add-Aspirin trial – a large, multicentre trial recruiting across a number of tumour types, where opportunities to co-enrol patients have been proactively explored and managed. The potential benefits of co-enrolment include: improving recruitment feasibility; increased opportunities for patients to participate in trials; and collection of robust data on combinations of interventions, which will ensure the ongoing relevance of individual trials and provide more cohesive evidence to guide the management of future patients. There are a number of perceived barriers to co-enrolment in terms of scientific, safety and ethical issues, which warrant consideration on a trial-by-trial basis. In many cases, any potential effect on the results of the trials will be negligible – limited by a number of factors, including the overlap in trial cohorts. Participant representatives stress the importance of autonomy to decide about trial enrolment, providing a compelling argument for offering co-enrolment where there are multiple trials that are relevant to a patient and no concerns regarding safety or the integrity of the trials. A number of measures are proposed for managing and monitoring co-enrolment. Ensuring acceptability to (potential) participants is paramount. Opportunities to enter patients into more than one cancer trial should be considered more routinely. Where planned and managed appropriately, co-enrolment can offer a number of benefits in terms of both scientific value and efficiency of study conduct, and will increase the opportunities for patients to participate in, and benefit from, clinical research.

## Statement of Search Strategies Used and Sources of Information

The paper largely reflects expert opinions and experiences of the authors, and their knowledge of the literature. The Pubmed database was searched for relevant articles, but a formal search strategy was not defined.

## Introduction

Co-enrolment – entering patients into more than one clinical trial either concurrently or sequentially – is rarely reported or discussed in oncology literature. As such, co-enrolment policies may be specified in the trial protocol or decisions made by an institute or recruiting investigator, without a clear rationale or evidence base. With a lack of guidance or consensus on when co-enrolment is appropriate, it is unsurprising that the decision not to co-enrol may be seen as the safe option.

Recent trends in oncology research – such as the use of longer term, maintenance therapies and evaluation of repurposed agents (whose use alongside other treatments may already be well documented) – as well as the ever-increasing number of trials competing for the same patients, mean that co-enrolment is becoming more relevant. More routine consideration of opportunities to enter patients into multiple trials is warranted.

Co-enrolment has been explored in other (non-cancer) settings – particularly those where trial recruitment is challenging and/or there are many (large) competing trials – including resuscitation [1], critical care [2], [3], [4] (including neonatal [5] and paediatric [6] settings) and peri-natal research [7]. Here, co-enrolment offers the opportunity to maximise use of the patient population and increase the speed and efficiency of research delivery. In settings such as HIV [8] and anaesthesia [9], where large, pragmatic trials are common and/or participants might be receiving several other medications, co-enrolment may also provide important data on drug interactions.

Across different settings, researchers report barriers to co-enrolment and, frequently, a lack of (universal) support from the research community or ethics committees [2], [6], [9]. Common barriers range from ethical and scientific considerations to safety concerns [1], [2], [3], [6], [7], [9]. The need for further reporting of co-enrolment and more research on this topic, is noted [4], [7], [10].

The potential benefits of co-enrolment, as well as possible barriers, are relevant in oncology trials and warrant further exploration. Here, we report our experiences with exploring and managing co-enrolment opportunities within a large, multicentre oncology trial.

## The Add-Aspirin Trial

**Fig 1:**
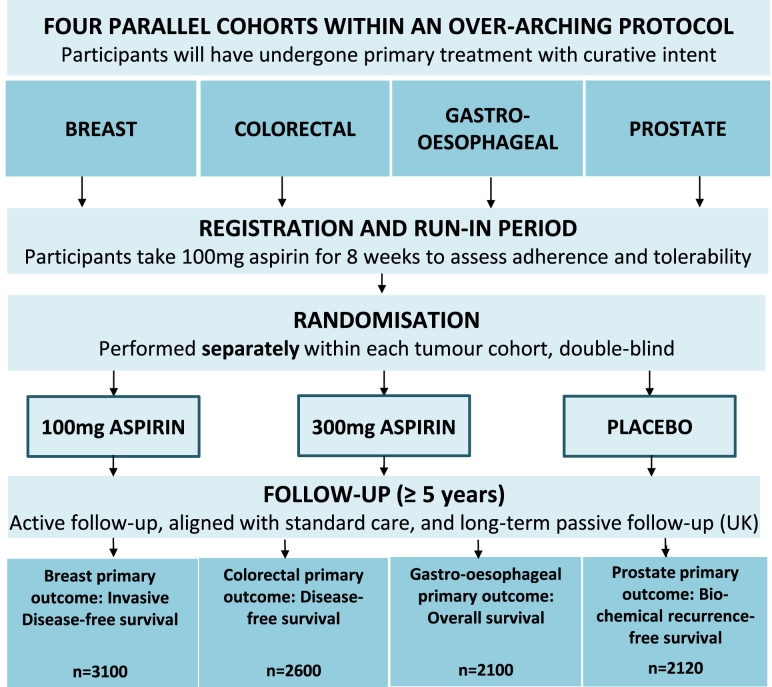
The Add-Aspirin trial.

The Add-Aspirin trial is a randomised controlled trial (RCT) assessing whether regular aspirin use after curative treatment for an early stage tumour can prevent recurrence and prolong survival (Figure 1) [11], [12], [13]. The intervention is being tested in four tumour types (breast, colorectal, gastro-oesophageal and prostate) by means of parallel cohorts. Patients enrol following potentially curative therapy – this incorporates a range of treatment pathways for each tumour site, including surgery with any appropriate (neo-)adjuvant therapies, radical chemoradiation (oesophageal) and radical radiotherapy (prostate). Participants are randomised to daily aspirin 100 mg, 300 mg or placebo. The trial is recruiting across the UK, and will also open in India, with a target of approximately 10 000 participants.

Co-enrolment may be relevant to patients entering Add-Aspirin subsequent to enrolling in a primary therapy trial and may also arise at the time of recurrence during participation in Add-Aspirin. A proactive approach to exploring co-enrolment opportunities with other trial teams has been adopted to agree when this might be appropriate and how it can be facilitated and managed within the ongoing trials.

## Benefits of Co-enrolment

Co-enrolment is particularly relevant in Add-Aspirin as the intervention is being given after initial treatment, so participants from trials of primary therapies represent a significant proportion of the eligible population. However, the potential advantages of co-enrolment apply more widely to multicentre oncology RCTs – particularly pragmatic trials – as a number of different interventions will be relevant to a patient over the course of their disease and treatment. Allowing appropriate co-enrolment improves the efficiency of recruitment, helping to ensure the feasibility of trials running concurrently, and maximises opportunities for patients to participate in, and benefit from, clinical research.

A further advantage is the opportunity to assess trial interventions alongside one another, helping to ensure the ongoing relevance of the studies. If two interventions might potentially both be given to a patient in future practice, collection of information on their combined use will be valuable for establishing the importance of each one, providing more cohesive evidence to inform the management of future patients.

There are, of course, potential concerns in allowing patients to enrol in multiple RCTs. In what follows, we consider the scientific, safety and ethical issues.

## Impact on Trial Results

A principle concern with co-enrolment is the potential effect on the results of the trials, particularly when they are evaluating a common outcome measure. We would argue that, although this issue deserves careful consideration, in many cases any effect will probably be negligible, and should not generally be a prohibitive factor.

In Add-Aspirin, due to the timing of the intervention, we are commonly considering the case of sequential co-enrolment – patients entering Add-Aspirin having previously enrolled in another trial. This would also be the case if considering trials of second- or third-line treatment after relapse in patients who had participated in a primary therapy trial. Here, assuming there is no interaction between the interventions, there is no concern about an effect on the results of the second trial (Add-Aspirin). However, there is the potential for an effect on the results of the first trial if participants from the different arms enter Add-Aspirin at different rates. This may occur because patients from one arm of the trial are either more likely to be eligible (for example, patients need to be disease-free, which may be more likely in the experimental arm of the first trial) or they are more likely to be willing to participate (for example, if one arm of the first trial has a shorter or less toxic treatment). In these scenarios, if aspirin is effective, it will have a differential effect in the trial arms of the first trial with the potential to affect the power. Although stratification within Add-Aspirin for the trial arm in the first trial will help to ensure balance in terms of those individuals entering Add-Aspirin, there may still be an overall imbalance in terms of aspirin allocation between the arms of the first trial when those who did not join Add-Aspirin are also considered.

**Fig 2:**
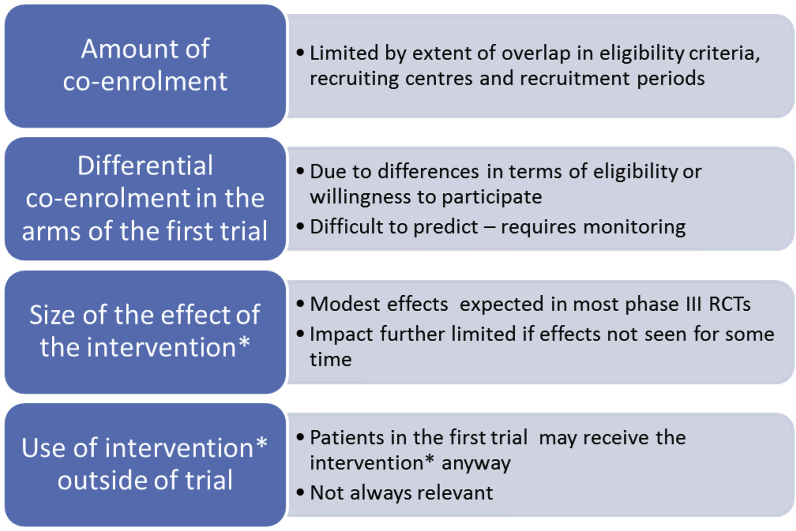
Factors affecting the potential effect of co-enrolment on power. *Intervention being evaluated in the second trial.

We have estimated the magnitude of any potential effect in different scenarios and found that it is generally limited by a number of factors (Figure 2). Statistical modelling, using ranges of assumptions, suggests that any effect on the power of the first trial will probably be small. Table A1 (web appendix) provides an example showing selected models, including some felt to illustrate the largest plausible effect. Significant effects were only anticipated with relatively large (improbable) differences in participation rates and were further increased when there were unexpectedly large effects of aspirin. Co-enrolling trials could be monitored for this unlikely set of circumstances, with the potential to stop co-enrolment if there were concerns. Our models do not consider the potential effect of aspirin use outside the Add-Aspirin trial (participants from the first trial already taking aspirin), which may further limit any effect. Similar limiting factors are noted in other settings [9]. However, this should be carefully considered on a trial-by-trial basis, before any co-enrolment, and subsequently monitored.

## Interaction Effects

Our modelling has generally assumed that there are no interaction effects between trial interventions – this is reasonable in most scenarios considered in relation to Add-Aspirin. However, others argue that the potential for an interaction between two trial interventions should not necessarily prohibit co-enrolment and, in fact, can facilitate evaluation of the interaction (particularly relevant where interventions are already in use outside of the trials) [7], [8], [9]. The information gained from co-enrolled participants may be insufficient to formally establish if there is an interaction but will be more robust data than would otherwise be available [7], [8], [9]. Modelling based on pragmatic anaesthesia trials suggested that a large detrimental effect on the power of the first trial would only be seen with a large antagonistic interaction, substantial co-enrolment and limited use of the second trial intervention outside of the trial [9].

A factorial randomisation can be viewed as preferable to co-enrolment between separate trials, but may not always be practical or sensible – for example, if the combination of the two interventions is only relevant to a small subgroup or, as with Add-Aspirin, one intervention is given at a later time, dictating the most appropriate timing for randomisation. Furthermore, the (statistical) advantages of a factorial design, compared with co-enrolment, may not be great [4], [8], [9]. The potential for loss of power for assessing one intervention, in the presence of the other, may still exist, and factorial trials are not normally powered to detect interactions.

## Safety Considerations

There may, of course, be safety concerns with patients receiving interventions from two different trials. This will be highly dependent on the interventions. If there are concerns or a high degree of uncertainty about toxicity risks then co-enrolment will probably be avoided. However, in a trial of a marketed product or intervention that is already in use in normal practice, co-enrolment will be more acceptable [9]. In Add-Aspirin, participants receive low-dose aspirin or placebo. In most of the primary treatment trials where co-enrolment may be relevant, there will already be patients taking aspirin alongside the trial intervention. Allowing participants to subsequently enrol in Add-Aspirin may facilitate the collection of more robust data on the use of aspirin alongside (or following) the intervention to guide future practice.

Concerns regarding liability, in the event of a personal injury claim being made by a trial participant who is enrolled in multiple trials, have been raised as a potential barrier to co-enrolment, but we do not believe this is justified. Existing indemnity arrangements for each trial should suffice.

## The Participant's Perspective

In addition to potential scientific benefits, allowing trial co-enrolment, where appropriate, will maximise opportunities for patients to participate in research. However, the approach must be both ethically sound and acceptable to (potential) participants – these are perhaps the most complex issues surrounding co-enrolment and there is currently a lack of guidance or evidence in the literature to inform this.

In our discussions with other trial teams regarding co-enrolment, some researchers have expressed concerns that asking patients to join more than one trial may over-burden them, a view that has proved to be a barrier in other settings [6]. The participant representatives on the Add-Aspirin Trial Management Group (co-authors on this paper), have been strong advocates of co-enrolment from the outset, and would argue that there is an opposing ethical obligation to provide all of the information required to allow an individual to decide for themselves about joining any trial that is relevant to them. Not approaching a patient to participate in a trial that they could be eligible for because they are already enrolled in another study would be denying them an opportunity. Similar conclusions were reached in a review of co-enrolment considerations in the anaesthesia setting, with the authors feeling that preventing patients from autonomously co-enrolling is difficult to justify ethically [9].

A survey of patients in a research-active breast cancer unit provides evidence to support these views [14]: three-quarters of respondents (37/50, 74%) would have considered entering more than one study if adequate written information was provided. Most (32/50, 64%) did not believe that participation in clinical research should be restricted to a maximum number of studies – and, of those who did, only two indicated that it should be limited to a single study. Furthermore, two-thirds of respondent (34/50, 68%) did not think that involvement in more than one study was a significant burden.

A similar survey of 50 families approached about multiple (up to six) clinical trials in a neonatal intensive care unit suggested similar attitudes (despite the setting, where the potential to over-burden families may be even more of a concern) [5]: three-quarters (74%) of parents indicated that they would enrol their baby into two or more studies; almost all (98%) felt they wanted to make the decisions about study enrolment themselves, rather than a clinician deciding.

Although the data from these studies are reassuring, they are limited and may reflect the views of select groups of individuals. Further exploration with patients and with groups representing patient and public involvement (PPI) is warranted.

## Measures to Increase Acceptability

Comments from respondents in the breast unit study emphasised the importance of individual choice as well as concerns around extra hospital visits interfering with normal life [14]. These are areas that need to be addressed in trials where co-enrolment is deemed appropriate.

Researchers have an obligation to carefully consider the timing of approaching potential participants about each trial, ensuring that the information provided (not only about the individual trials but also about the implications of joining more than one) is clear and there is sufficient opportunity for questions. In the paediatric intensive care setting, Harron *et al*. [6] advocated careful development and piloting of a strategy for the whole consent process when multiple studies may be available to an individual.

Wherever possible, there should be compatibility between follow-up schedules for two trials where co-enrolment is possible in order to minimise the number of additional hospital visits and assessments/tests compared with standard care. Ideally, this would be planned at the design stage. Where co-enrolment decisions may be made during the trial, allowing some flexibility in schedules will work towards this aim – enabling research nurses to plan clinic visits that will meet the requirements of both trial schedules. In Add-Aspirin, follow-up schedules have been planned to largely align with standard care – and this will be the case with many pragmatic trials. Additionally, there is some flexibility regarding the timing of assessments.

In the above considerations, engagement with and input from participant representatives and PPI groups is vital to ensure that the approach is acceptable to participants and will not lead to unnecessary additional burden.

## Ethical Approvals

Some researchers report resistance from ethics committees as an obstacle to allowing individuals to enter multiple trials [2], [14]. This has not been the experience in Add-Aspirin – the potential for participants from multiple primary treatment trials to enrol in Add-Aspirin has been written into the trial protocol from the outset, and was not raised as an issue by the ethics committee who approved the study, nor by the regulators nor funders of the trial. Thus, there is perhaps a need for a more consistent approach to trial co-enrolment by research ethics committees. We would suggest that co-enrolment, where appropriate, should generally be supported in order to allow potential participants the autonomy to decide about enrolling in any trial that is relevant to them. However, this should be on the provisos that: the informed consent process and trial follow-up schedules have been carefully considered; the safety of receiving both trial interventions has been deemed acceptable; and any other appropriate measures are in place to minimise any extra burden on participants as far as possible.

The potential scientific advantages of allowing co-enrolment (where appropriate), in terms of increasing both the value and efficiency of the research, provide further ethical justification for the approach. Myles *et al*. [9] argue that an important ethical consideration in research planning is the efficient conduct of studies and fairer allocation of resources for research, and that allowing co-enrolment can contribute to this aim. Furthermore, if two interventions being evaluated in trials might potentially both be given to a patient in future practice, there is arguably an ethical obligation for researchers to collect information on the combined use of the therapies in order to establish the importance and safety of each one in the context of the other, and provide more cohesive evidence to inform the management of future patients [7].

## Managing Co-enrolment

Where co-enrolment to multiple oncology RCTs is permitted, given the issues outlined here, it requires careful management and monitoring. We propose a number of measures (Table 1).

**Table 1:** Proposed measures for trial teams managing co-enrolment within a randomised controlled trial

	Proposed measure	Purpose
Design	Identify trials where co-enrolment may be considered	Assess potential impact and agree where co-enrolment is appropriate in advance

	Develop appropriate consent process∗	Ensure that being approached about multiple studies will be acceptable to patients

	Ensure compatibility of follow-up schedules, allowing flexibility where possible/appropriate∗	Minimise extra visits/assessments, ensuring that participation in multiple studies will be acceptable to patients

	Provide guidance on co-enrolment in the protocol (and trial website/other documents as appropriate)	Ensure only appropriate co-enrolment takes place and follows the strategy developed for consent and follow-up

	Consider stratifying by treatment arm in the first trial in the randomisation algorithm for the second trial (where significant overlap is expected)	Ensure treatment allocation in the second trial is balanced (in terms of those individuals who *enter* the second trial)†

Conduct	Implement eligibility checks around co-enrolment at entry	Ensure only appropriate co-enrolment takes place

	Consider implementation of screening logs	Identify any recruitment issues as a result of co-enrolment decisions or any barriers to co-enrolment

Monitoring	Collect and regularly review co-enrolment information, including treatment allocation in the other trial, on case report forms	Active monitoring with the potential to take action – by capping recruitment from one arm, for example – if a large imbalance occurs (although this is unlikely)

	Establish agreements to share information between data monitoring committees (blinded trials)	It may be appropriate for monitoring to be carried out by data monitoring committees in the case of blinded trials


∗ In discussion with participant representatives and/or patient and public involvement groups.

† This will not ensure balance overall if participants from the different treatment arms of the first trial enter the second trial at different rates. Thus, careful monitoring is still required.

A precedent for designing and conducting RCTs to facilitate co-enrolment has been set in the HIV field (Terry Beirn Community Programs for Clinical Research on AIDS; CPCRA) [8]. Measures include shared data collection forms; standardised definitions and criteria for assessing and reporting outcomes and adverse events; a single, common follow-up schedule; and an analysis approach that explores drug interactions.

## Discussion

For the vast majority of trials where co-enrolment with Add-Aspirin has been considered, we have found that it is likely to be acceptable both in terms of the safety of participants and maintaining the integrity of trial results. As such, the importance of giving individuals the autonomy to make their own decisions about trial participation provides a compelling ethical argument for allowing co-enrolment, wherever appropriate, providing that it is done in a way that will be acceptable to participants. We have encountered a number of perceived barriers that may not be well-founded and there is a need for further evidence to promote greater understanding about the potential impact of co-enrolment.

The benefits of co-enrolment align with the original aims in establishing the National Cancer Research Network, which include improving the co-ordination and quality of research, widening participation, increasing the numbers of patients involved and speeding up the delivery of research for the ultimate benefit to patients [15]. As such, we suggest it should be routinely considered by the associated clinical studies groups in reviewing trial portfolios, with the aim of maximising co-enrolment opportunities.

The implications of individuals participating in more than one trial are multifactorial and should be carefully considered on a trial-by-trial basis. Evidence relating to acceptability is limited, and more research is needed. However, as it is probably highly dependent on the patient group and the specific trials under consideration, engagement with relevant PPI groups and representatives, from the planning stage and throughout the trial, is crucial to ensure that the appropriate measures are in place. There is an onus on the trial teams to evaluate acceptability and any potential (scientific) consequences in advance, and to monitor co-enrolment closely throughout the trial, managing any issues appropriately with a pre-defined strategy. Trial protocols should not enforce a complete ban on co-enrolment without sound justification.

Much could be learnt from the CPCRA programme, where efforts to facilitate co-enrolment led to a quarter (22.5%) of patients from six RCTs entering more than one trial [8]. The programme was developed by a single research group – a high degree of co-operation and strong lines of communication would be required to achieve similar where trials are being conducted by different groups. In other settings, the establishment of co-enrolment policies or consensus guidelines has been advocated [4], [8], [10]. This could be a way forward in oncology research.

A more considered and co-operative approach to co-enrolment will not only benefit individual trials, but may contribute to an evidence base showing the extent of co-enrolment and any observed impact or issues. Limited reports from other settings have not indicated any negative impact [3], [6]. Based on the experiences in Add-Aspirin, we hope that such data might ultimately reassure researchers of the benefits of allowing participants to co-enrol where there are multiple oncology trials that are relevant to them. Add-Aspirin opened in October 2015 and, to date, the possibility of co-enrolling relevant patients has been agreed for 40 other trials, across the four tumour types. The number of participants who have been co-enrolled remains small at this early stage of recruitment.

## Conclusions

Opportunities for co-enrolment of participants into multiple cancer trials should be more routinely considered. Where planned and managed appropriately, co-enrolment can offer a number of benefits in terms of both the scientific value and efficiency of study conduct, and will increase the opportunities for patients to participate in, and benefit from, clinical research.

## Figures and Tables

**Table A1:** Example power calculations to assess the potential impact of co-enrolment

Modelling assumptions			Estimated impact on trial X				
Effect of aspirin on 5 year survival∗	Participation rates in Add-Aspirin†		5 year survival in trial X with co-enrolment			Power (loss/gain in power)	Extra patients (OR follow-up) needed for 80% power
	Control	Intervention	Control	Intervention	Difference		
Trial X result is positive (5 year survival 55% control versus 45% intervention) in the absence of co-enrolment							
6%	10%	10%	45.4%	55.4%	10.0%	79.9%	2 (1 month)
	20%	20%	45.8%	55.8%	10.0%	79.9%	3 (1 month)
	30%	30%	46.2%	56.2%	10.0%	79.8%	5 (1 month)
	10%	15%	45.4%	55.6%	10.2%	81.5%	–
	10%	20%	45.4%	55.8%	10.4%	82.9%	–
	15%	30%	45.6%	56.2%	10.6%	84.3%	–
	15%	10%	45.6%	55.4%	9.8%	78.3%	44 (3 months)
	20%	10%	45.8%	55.4%	9.6%	76.6%	89 (5 months)
	30%	15%	46.2%	55.6%	9.4%	74.8%	138 (8 months)
10%	10%	10%	45.7%	55.7%	10.0%	79.9%	3 (1 month)
	20%	20%	46.3%	56.3%	10.0%	79.8%	5 (1 month)
	30%	30%	47.0%	57.0%	10.0%	79.8%	7 (1 month)
	10%	15%	45.7%	56.0%	10.3%	82.4%	–
	10%	20%	45.7%	56.3%	10.7%	84.7%	–
	15%	30%	46.0%	57.0%	11.0%	86.8%	–
	15%	10%	46.0%	55.7%	9.7%	77.2%	75 (5 months)
	20%	10%	46.3%	55.7%	9.3%	74.2%	154 (9 months)
	30%	15%	47.0%	56.0%	9.0%	71.1%	244 (15 months)
Trial X result is negative (5 year survival 45% in both arms) in the absence of co-enrolment							
6%	10%	10%	45.4%	45.4%	0.0%		
	20%	20%	45.8%	45.8%	0.0%		
	30%	30%	46.2%	46.2%	0.0%		
	10%	15%	45.4%	45.6%	0.2%		
	10%	20%	45.4%	45.8%	0.4%		
	15%	30%	45.6%	46.2%	0.6%		
	15%	10%	45.6%	45.4%	−0.2%		
	20%	10%	45.8%	45.4%	−0.4%		
	30%	15%	46.2%	45.6%	−0.6%		
Trial X result is negative (5 year survival 45% in both arms) in the absence of co-enrolment							
10%	10%	10%	45.7%	45.7%	0.0%		
	20%	20%	46.3%	46.3%	0.0%		
	30%	30%	47.0%	47.0%	0.0%		
	10%	15%	45.7%	46.0%	0.3%		
	10%	20%	45.7%	46.3%	0.7%		
	15%	30%	46.0%	47.0%	1.0%		
	15%	10%	46.0%	45.7%	−0.3%		
	20%	10%	46.3%	45.7%	−0.7%		
	30%	15%	47.0%	46.0%	−1.0%		

The table illustrates the potential impact of co-enrolment into Add-Aspirin on the power of a hypothetical study, trial X. Selected results are shown from models performed under a range of assumptions about the factors listed in Figure 2, including scenarios felt to illustrate the largest plausible impact on power.

Trial X: A hypothetical two-arm superiority randomised controlled trial of a new peri-operative chemotherapy regimen versus standard in gastro-oesophageal patients. Designed with 80% power to detect a 10% improvement (from 45% to 55%) in survival at 5 years, requiring 500 patients per arm. Patients who are disease free at the end of treatment may become eligible for Add-Aspirin.

∗ A 6% survival benefit at 5 years is hypothesised in Add-Aspirin (gastro-oesophageal). Models are repeated for larger benefits to illustrate potential effects on power.

† A range of participation rates are used to assess potential impact – actual rates are unlikely to reach 30% (limited by overlap in recruiting centres and recruitment periods, as well as trial X participants being ineligible or unwilling to participate in Add-Aspirin). Differences in rates between arms are also unlikely to be as large as illustrated here.
